# Clinical Characteristics and Prognostic Analysis of Patients With Pulmonary Tuberculosis and Type 2 Diabetes Comorbidity in China: A Retrospective Analysis

**DOI:** 10.3389/fpubh.2021.710981

**Published:** 2021-08-25

**Authors:** Shijie Zhang, Xiang Tong, Lei Wang, Tianli Zhang, Jizhen Huang, Dongguang Wang, Lian Wang, Hong Fan

**Affiliations:** Department of Respiratory and Critical Care Medicine, West China Hospital/West China School of Medicine, Sichuan University, Chengdu, China

**Keywords:** clinical characteristics, prognostic analysis, pulmonary tuberculosis, type 2 diabetes, all-cause death

## Abstract

**Background:** Tuberculosis (TB) is one of the leading communicable diseases, with significant morbidity and mortality. Diabetes can increase the risk of developing TB and the related adverse outcomes. This study retrospectively analyzed the clinical characteristics and prognosis of patients with pulmonary TB and type 2 diabetes comorbidity.

**Methods:** About 282 cases with pulmonary TB and type 2 diabetes comorbidity were identified from West China Hospital between January 1, 2010, and December 31, 2016, and were followed up for at least 3 years. We further used Kaplan–Meier methods and COX regression analysis to identify the influence factors for all-cause death.

**Results:** Compared to the survival patients, patients who died were older, exhibited significantly lower albumin and hemoglobin levels, but higher Charlson Comorbidity Index (CCI) score at admission, and had a lower usage rate of metformin. The all-cause mortality rates at 1 and 5 years were 5.67 and 20.59%, separately. For 1-year all-cause death, higher albumin level (HR = 0.90, 95% CI: 0.81–0.99) was the independently protective factor, but older age (HR = 1.07, 95% CI: 1.01–1.13) and CCI score ≥3 (HR = 6.77, 95% CI: 1.40–32.69) were the independent risk factors. For long-term all-cause death, higher albumin level (HR = 0.94, 95% CI: 0.88–1.00), the use of metformin (HR = 0.21, 95% CI: 0.07–0.59), insulin (HR = 0.27, 95% CI: 0.10–0.74), or sulfonylureas (HR = 0.23, 95% CI: 0.07–0.74) were the independently protective factors, but older age (HR = 1.03, 95% CI: 1.00–1.07) and CCI score ≥3 (HR = 7.15, 95% CI: 2.56–19.92) were the independent risk factors.

**Conclusions:** The lower albumin level, older age, and CCI score ≥3 were predictors of all-cause death in patients with pulmonary TB and type 2 diabetes comorbidity. In the long run, patients who use metformin, insulin, or sulfonylureas as hypoglycemic agents may have a lower incidence of death.

## Introduction

Tuberculosis (TB) is one of the leading communicable diseases caused by *Mycobacterium tuberculosis* (Mtb), with 10.0 million new cases and 1.4 million deaths in 2019 globally (1). In the TB epidemiological sampling survey in 2010 in China, there were an estimated 4.99 million active TB patients, which was decreasing by ~0.2% per year from 2000 (2). However, this progress is threatened by the global demographic transition resulting in a rapid rise in the burden of type 2 diabetes mellitus (3), particularly in low-income and middle-income countries with the highest burden of TB. There is strong evidence that diabetes increases the risk of TB by two to three times. Modeling studies have shown that a 25% increase in diabetes prevalence worldwide would offset the present downward trajectory in global TB incidence by 8% by 2035 (4).

Diabetes not only increases the risk of developing TB, but also increases the related adverse outcomes. Nguyen et al. reported that diabetes could increase the all-cause mortality among patients during anti-TB treatment in California (5), and similarly, Ko et al. found that diabetes could increase 1-year all-cause mortality among newly diagnosed TB patients in an Asian population (6). A meta-analysis including 104 studies also showed that diabetes was associated with increased risks of mortality and might increase the risk of developing primary multidrug-resistant TB (7). Moreover, diabetes could also increase the risk of treatment failure and recurrence in TB patients (8). So reasonable blood glucose management in patients with TB and type 2 diabetes comorbidity may bring unexpected benefits, and the choice of diabetes medications is particularly important. In this retrospective study, we aimed to analyze the clinical characteristics of patients with pulmonary TB and type 2 diabetes comorbidity by focusing on the clinical features, laboratory findings, different diabetes medications, and the outcome of death, and thereby shed light on the treatment of this disease.

## Materials and Methods

### Study Population

We included the hospitalized patients (≥18 years old) with a diagnosis of pulmonary TB and type 2 diabetes of West China Hospital in Chengdu (China) between January 1, 2010, and December 31, 2016. Type 2 diabetes must be diagnosed before or at the same time as pulmonary TB. The exclusion criteria were as follows: (1) patients with severe infections, metabolic disorders, consciousness disturbances, acute liver failure, acute heart failure, and acute respiratory failure; (2) patients with chronic kidney disease (>3b stage); (3) patients with type 1 diabetes; (4) patients with TB and (or) diabetes without the formal treatment; and (5) patients with diagnostic anti-TB. The study was approved by the Ethics Committee of West China Hospital of Sichuan University (2019-1103).

### Data Collection

The History Information System (HIS) was queried to identify all patients with pulmonary TB and type 2 diabetes comorbidity. If a patient had multiple records of hospitalization between 2010 and 2016, the record in which the patient was first diagnosed with pulmonary TB was used for further analysis. Data extracted from the HIS included demographic information, clinical parameters, underlying diseases, and medications for diabetes, and these data were from the first 3 days of hospitalization before anti-TB drugs and other related drugs were used. For hypoglycemic medication, only those continuously used for more than 3 years after the diagnosis of pulmonary TB were considered the effective use of hypoglycemic drugs, and the others were used as negative controls. Biochemical variables included albumin, hemoglobin, alanine transaminase, fasting plasma glucose (FPG), glycated hemoglobin (HbA1c), and serum creatinine. Pulmonary cavitation was identified by the image of chest computed tomography. Charlson Comorbidity Index (CCI) scores were calculated for every patient included in the study population to quantify the degree of underlying comorbidity at the time of diagnosis of pulmonary TB (9, 10), but in our study, pulmonary TB and type 2 diabetes were not included in the CCI score system. All included patients were followed up to obtain their survival data. The last follow-up time of this study was December 31, 2019.

### Definitions

The study end point was all-cause death. The survival time was calculated as the number of days between the initiation of anti-TB therapy and death. The definition of pulmonary TB depended on the Chinese guidelines for the diagnosis and treatment of TB (2001 version) (11): (1) The sputum smear was positive for acid-fast bacilli; (2) the nucleic acid of Mtb and/or Mtb culture was positive in sputum, bronchoalveolar lavage fluid, or pleural effusion; (3) the pathological staining of lung tissue specimens was positive for acid-fast bacilli, or the nucleic acid of Mtb was positive in lung tissue specimens from the lesion site. The diagnosis of pulmonary TB could be clarified if any of the above three items were met. The definition of cure for pulmonary TB is treatment completed with the disappearance of symptoms, improvement of lung lesions in chest computed tomography, and acid-fast bacilli smears or cultures with negative results at the end of therapy. Type 2 diabetes was defined as diabetic symptoms plus random blood glucose ≥11.1 mmol/L, and/or FPG ≥7.0 mmol/L, and/or blood glucose in 2 h after oral glucose tolerance test ≥11.1 mmol/L (12).

### Statistical Analysis

The statistical analyses were performed using software SPSS 21.0 software (SPSS, Chicago, IL, United States) and GraphPad Prism version 7 software (Graph software, San Diego, CA, United States). A *p* < 0.05 was considered statistically significant, and all statistical analyses were two-sided. The continuous variables with normal distribution were expressed as mean ± standard deviation, and Student's *t*-test was used for comparisons between two groups. The continuous variables with non-normal distribution were described as median (25th percentile, 75th percentile), and the rank-sum test was used for comparisons among groups. Frequency and percentage were for categorical variables, which were analyzed by χ2 test. Ordinal data were analyzed by the rank-sum test. Kaplan–Meier survival curves and Cox regression analysis (“Enter” method) were used to identify the influence factors associated with all-cause death in patients with pulmonary TB and type 2 diabetes comorbidity, and significance in survival curves was determined using the log-rank test.

## Results

### General Characteristics

#### Study Population

**Figure 1:**
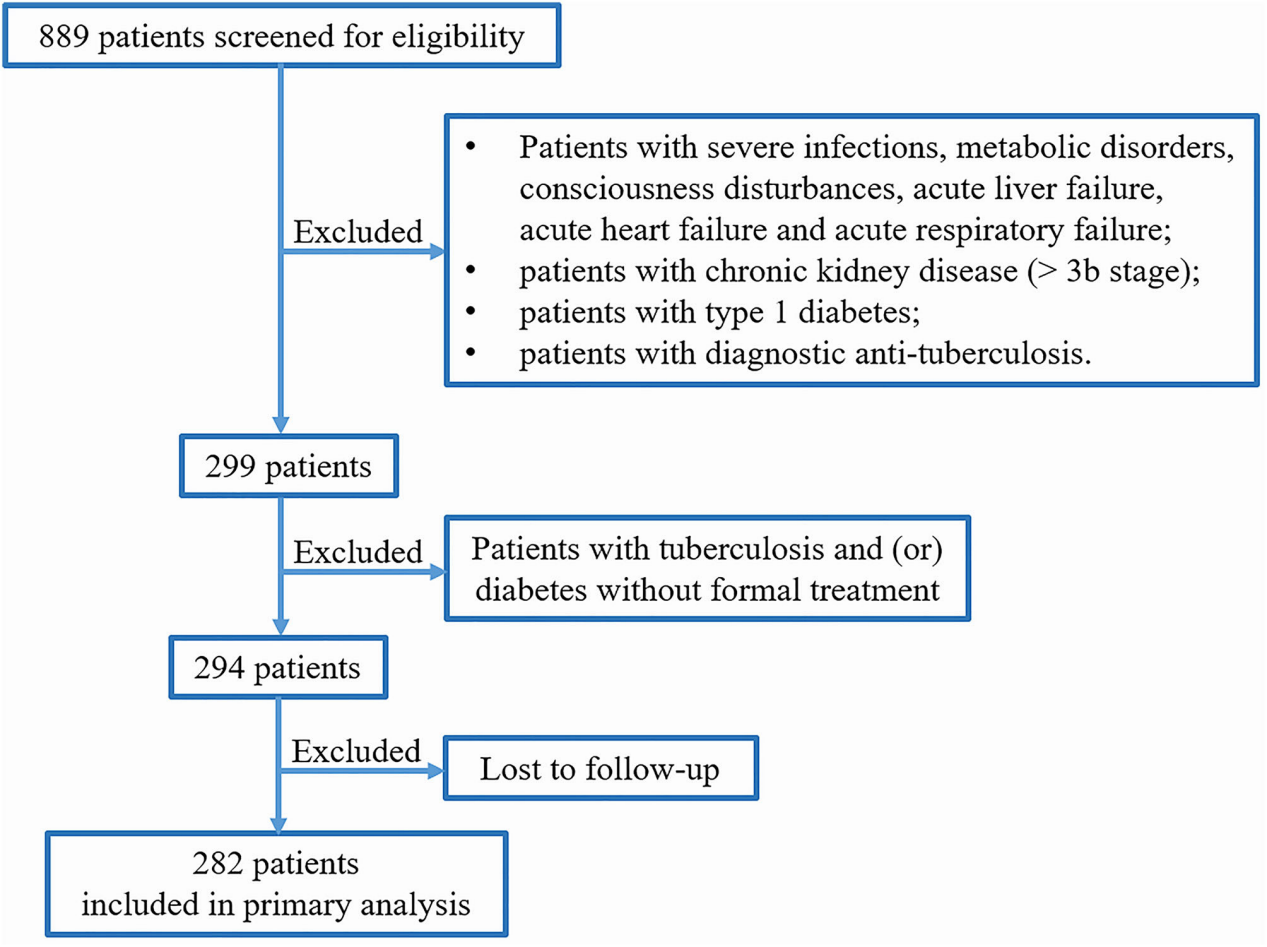
Flow diagram of the selection of patients for inclusion.

Of 889 patients screened, 294 were included, of whom 12 were subsequently lost to follow up, leaving 282 patients in the primary analysis population (Figure 1). Approximately 90% of the excluded patients were excluded for the reason of diagnostic anti-TB treatment (i.e. there was no pathogenic evidence of Mtb infection). All patients were followed up for at least 3 years, and 67.50% patients have been followed up for 5 years in addition to the deaths. During our follow-up, 42 patients died (66.67% of deaths were related to pulmonary TB), and the mortality was 14.89%. The average age was 57.15 years, and the patients who died were older overall (*P* = 0.004, Table 1). There were similar gender distribution and smoking rate in the survivors and non-survivors.

**Table 1:** The demographic and clinical parameters of patients with pulmonary TB and type 2 diabetes comorbidity at baseline visit.

	**Survivors**	**Non-survivors**	**Total**	***P*-value**
No. of participants	240 (85.11%)	42 (14.89%)	282	
Male, *n* (%)	190 (79.17%)	37 (88.10%)	227 (80.50%)	0.18
Age (years)	56.28 ± 11.82	62.1 ± 12.83	57.15 ± 12.13	0.004
Smoking, *n* (%)	145 (60.42%)	29 (69.05%)	174 (61.70%)	0.29
Pulmonary cavitation, *n* (%)	146 (60.83%)	23 (54.76%)	169 (59.93%)	0.46
Albumin (g/L)	36.02 ± 6.04	31.60 ± 6.70	35.36 ± 6.33	<0.001
Hemoglobin (g/L)	124.21 ± 20.48	115.29 ± 21.07	122.88 ± 20.78	0.01
Alanine transaminase (IU/L)^*^	19.50 (13.00, 31.75)	17.00 (12.00, 31.75)	19.00 (13.00, 31.25)	0.57
FPG (mmol/L)^*^	9.01 (6.89, 12.32)	7.79 (6.29, 10.26)	8.92 (6.82, 12.14)	0.04
HbA1c (%)	9.46 ± 2.36	8.74 ± 2.58	9.36 ± 2.39	0.19
Serum creatinine (μmol/L)^*^	67.30 (56.00, 76.55)	63.30 (53.58, 74.25)	67.00 (55.68, 76.10)	0.33
**Underlying disease**, ***n*****(%)**				
Myocardial infarction	2 (0.83%)	2 (4.76%)	4 (1.42%)	0.11
Cerebrovascular disease	12 (5.00%)	2 (4.76%)	14 (4.96%)	1.00
Chronic kidney disease	7 (2.92%)	1 (2.38%)	8 (2.84%)	1.00
Chronic liver disease	18 (7.50%)	3 (7.14%)	21 (7.45%)	1.00
Chronic lung disease	21 (8.75%)	13 (30.95%)	34 (12.06%)	<0.001
Solid tumor	8 (3.33%)	5 (11.90%)	13 (4.61%)	0.04
Hematological tumor	0	1 (2.38%)	1 (0.35%)	0.15
Autoimmune disease	8 (3.33%)	4 (9.52%)	12 (4.26%)	0.16
Ulcerative disease	4 (1.67%)	1 (2.38%)	5 (1.77%)	0.56
Peripheral vascular disease	5 (2.08%)	0	5 (1.77%)	1.00
HIV	1 (0.42%)	2 (4.76%)	3 (1.06%)	0.06
CCI score, *n* (%)^**^				<0.001
0	167 (69.58%)	18 (42.86%)	185 (65.60%)	
1–2	68 (28.33%)	16 (38.10%)	84 (29.79%)	
≥3	5 (2.08%)	8 (19.05%)	13 (4.61%)	
**Taking diabetes drug**, ***n*****(%)**				
Metformin	90 (37.50%)	7 (16.67%)	97 (34.40%)	0.01
Insulin	149 (62.08%)	26 (61.90%)	175 (62.06%)	0.98
Sulfonylureas	54 (22.50%)	4 (9.52%)	58 (20.57%)	0.06
Glinides	10 (4.17%)	3 (7.14%)	13 (4.61%)	0.65
α-Glucosidase inhibitor	50 (20.83%)	10 (23.81%)	60 (21.28%)	0.66
Thiazolidinediones	5 (2.08%)	0	5 (1.77%)	1.00
DPP-IV inhibitor	1 (0.42%)	0	1 (0.35%)	1.00
TB outcome (cure)^***^	88 (70.97%)	9 (32.14%)	97 (63.82%)	<0.001

*FPG, fasting plasma glucose; DPP-IV inhibitor, dipeptidyl peptidase-IV inhibitor*.

* *Data are not conform to the normal distribution, which was analyzed with the rank-sum test*.

** *Ordinal data were analyzed by the rank-sum test*.

*** *There were 124 and 28 patients in survivors and non-survivors for the outcome analysis of pulmonary TB*.

#### Laboratory Findings and Imaging Features

Relevant laboratory data and imaging features in the survivors and non-survivors are shown in Table 1. The average albumin level in all patients was 35.36 g/L, which was significantly below the lower limit of the normal range (40–55 g/L). Patients in the death group exhibited significantly lower albumin (31.60 vs. 36.02 g/L, *P* < 0.001) and hemoglobin (115.29 vs. 124.21 g/L, *P* = 0.01) at admission as compared with the survival group. There were no differences in alanine transaminase, HbA1c, and serum creatinine at the beginning of the study. Radiologically, the incidence of pulmonary cavitation was similar between the two groups.

#### Underling Diseases

As shown in Table 1, the most common underlying diseases in patients with pulmonary TB and type 2 diabetes comorbidity were chronic lung disease (12.06%), followed by chronic liver disease (7.45%), cerebrovascular disease (4.96%), solid tumor (4.61%), and autoimmune disease (4.26%). Patients in the death group had a higher incidence of underlying chronic lung disease (30.95 vs. 8.75%, *P* < 0.001) and solid tumor (11.90 vs. 3.33%, *P* = 0.04) than in the survival group. The CCI score in the death population was significantly higher as compared with the survival population (*P* < 0.001).

#### Diabetes Medications

All patients were taking one or more hypoglycemic drugs. The most commonly used diabetes drug was insulin (62.06%), followed by metformin (34.40%), α-glucosidase inhibitor (21.28%), and sulfonylureas (20.57%). Patients in the survival group had a higher usage rate of metformin than in the death group (37.50 vs. 16.67%, *P* = 0.01).

### Correlations Between 1-Year All-Cause Death and Clinical Parameters of Patients With Pulmonary TB and Type 2 Diabetes Comorbidity

The 1-year all-cause mortality was 5.67% (16/282) in patients with pulmonary TB and type 2 diabetes comorbidity. The univariate COX regression analysis revealed that age (HR = 1.07, 95% CI: 1.02–1.12, *P* = 0.003), albumin (HR = 0.90, 95% CI: 0.83–0.97, *P* = 0.01), and CCI score≥3 (HR = 9.31, 95% CI: 2.72–31.80, *P* < 0.001) were significantly correlated with the 1-year all-cause death. After applied multivariate COX regression analysis, we found that the higher albumin level (HR = 0.90, 95% CI: 0.81–0.99, *P* = 0.03) was the independently protective factor, but older age (HR = 1.07, 95% CI: 1.01–1.13, *P* = 0.02) and CCI score≥3 (HR = 6.77, 95% CI: 1.40–32.69, *P* = 0.02) were the independent risk factors for 1-year all-cause death in patients with pulmonary TB and type 2 diabetes comorbidity (Table 2).

**Table 2:** COX regression analysis of 1-year all-cause death in patients with pulmonary TB and type 2 diabetes comorbidity.

**Parameters**	**Univariate COX regression analysis**		**Multivariate COX regression analysis**	
	**HR (95% CI)**	***P*-value**	**HR (95% CI)**	***P*-value**
**Gender**				
Female	Reference		Reference	
Male	1.05 (0.30–3.68)	0.94	0.54 (0.09–3.23)	0.50
Age	1.07 (1.02–1.12)	0.003	1.07 (1.01–1.13)	0.02
Smoking	1.04 (0.38–2.86)	0.94	1.79 (0.45–7.06)	0.41
Pulmonary cavitation	0.52 (0.19–1.39)	0.19	0.91 (0.27–3.03)	0.87
Albumin	0.90 (0.83–0.97)	0.01	0.90 (0.81–0.99)	0.03
Hemoglobin	0.99 (0.97–1.01)	0.38	1.02 (0.99–1.05)	0.27
Alanine transaminase	1.00 (0.97–1.02)	0.64	0.99 (0.97–1.02)	0.53
Serum creatinine	1.01 (0.98–1.03)	0.62	1.01 (0.99–1.04)	0.31
**CCI score**				
0	Reference		Reference	
1–2	1.60 (0.51–5.03)	0.42	0.86 (0.25–2.96)	0.81
≥3	9.31 (2.72–31.80)	<0.001	6.77 (1.40–32.69)	0.02
**Taking diabetes drug**				
Metformin	0.43 (0.12–1.52)	0.19	0.30 (0.06–1.58)	0.16
Insulin	1.01 (0.37–2.77)	0.99	0.32 (0.06–1.84)	0.20
Sulfonylureas	0.54 (0.12–2.39)	0.42	0.39 (0.07–2.34)	0.31
α-glucosidase inhibitor	0.87 (0.25–3.04)	0.82	0.14 (0.02–1.00)	0.05

*For 1-year all-cause death, there were 16 deaths of all the 282 eligible patients*.

### Correlations Between Long-Term All-Cause Death and Clinical Parameters of Patients With Pulmonary TB and Type 2 Diabetes Comorbidity

**Figure 2:**
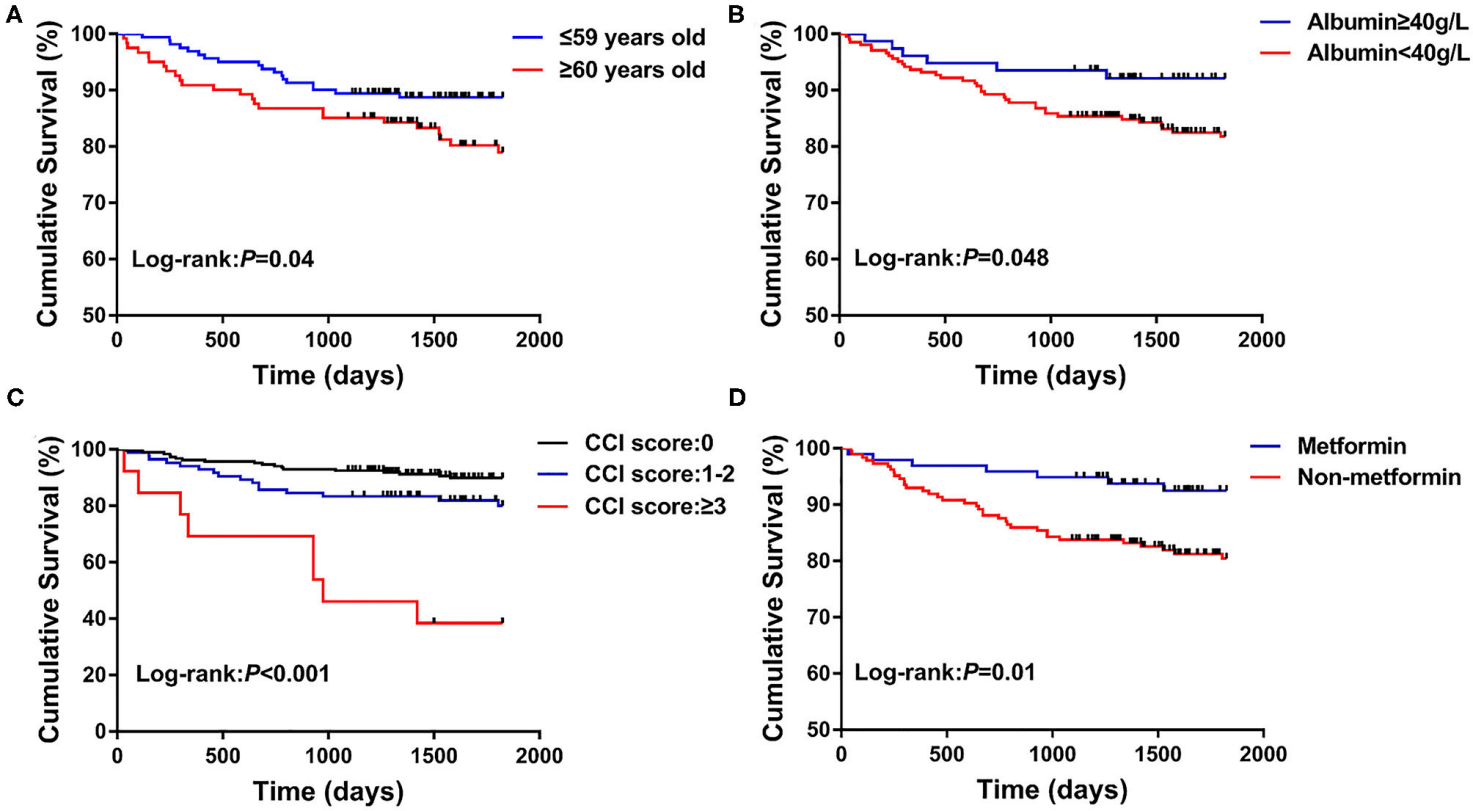
Kaplan–Meier curves of the long-term survival according to age category **(A)**, albumin category **(B)**, CCI score **(C)**, and the use of metformin **(D)**.

The median follow-up time was 1,825 days, and the all-cause mortality was 14.89% (42/282). About 204 patients were followed for 5 years, and the 5-year all-cause mortality was 20.59%. Univariate COX regression analysis revealed that age (HR = 1.04, 95% CI: 1.01–1.07, *P* = 0.003), albumin (HR = 0.90, 95% CI: 0.86–0.95, *P* < 0.001), hemoglobin (HR = 0.98, 95% CI: 0.97–1.00, *P* = 0.01), CCI score 1-2 (HR = 2.08, 95% CI: 1.06–4.07, *P* = 0.03), CCI score≥3 (HR = 8.72, 95% CI: 3.78–20.11, *P* < 0.001), and the use of metformin (HR = 0.36, 95% CI: 0.16–0.81, *P* = 0.01) were significantly correlated with the all-cause death. After the applied multivariate COX regression analysis, age (HR = 1.03, 95% CI: 1.00–1.07, *P* = 0.03), albumin (HR = 0.94, 95% CI: 0.88–1.00, *P* = 0.048), CCI score≥3 (HR = 7.15, 95% CI:2.56–19.92, *P* < 0.001), and the use of metformin (HR = 0.21, 95% CI: 0.07–0.59, *P* = 0.003) remained independently associated with all-cause death, but insulin (HR = 0.27, 95% CI: 0.10–0.74, *P* = 0.01) and sulfonylureas (HR = 0.23, 95% CI: 0.07–0.74, *P* = 0.01) were also independently associated with all-cause death (Table 3). In particular, higher albumin level, metformin, insulin, and sulfonylureas were the independently protective factors, but older age and CCI score≥3 were the independent risk factors for all-cause death in patients with pulmonary TB and type 2 diabetes comorbidity. In addition, the cure of pulmonary TB was also a protective factor for long-term all-cause death in patients with pulmonary TB and type 2 diabetes comorbidity (*P* < 0.001). Kaplan–Meier curves of overall patient survival according to age category, albumin category, CCI score, and the use of metformin are shown in Figure 2.

**Table 3:** COX regression analysis of the long-term all-cause death in patients with pulmonary TB and type 2 diabetes comorbidity.

**Parameters**	**Univariate COX regression analysis**		**Multivariate COX regression analysis**	
	**HR (95% CI)**	***P*-value**	**HR (95% CI)**	***P*-value**
**Gender**				
Female	Reference		Reference	
Male	1.79 (0.70–4.56)	0.22	1.41 (0.43, 4.64)	0.57
Age	1.04 (1.01–1.07)	0.003	1.03 (1.00–1.07)	0.03
Smoking	1.39 (0.72–2.68)	0.32	1.72 (0.74, 3.98)	0.21
Pulmonary cavitation	0.79 (0.43–1.44)	0.44	1.20 (0.57–2.54)	0.64
Albumin	0.90 (0.86–0.95)	< 0.001	0.94 (0.88–1.00)	0.048
Hemoglobin	0.98 (0.97–1.00)	0.01	1.00 (0.98–1.02)	0.88
Alanine transaminase	1.00 (0.98–1.01)	0.54	0.99 (0.98–1.01)	0.27
Serum creatinine	0.99 (0.98–1.01)	0.39	1.00 (0.98–1.02)	0.82
**CCI score**				
0	Reference		Reference	
1–2	2.08 (1.06–4.07)	0.03	1.29 (0.62–2.69)	0.49
≥3	8.72 (3.78–20.11)	<0.001	7.15 (2.56–19.92)	<0.001
**Taking diabetes drug**				
Metformin	0.36 (0.16–0.81)	0.01	0.21 (0.07–0.59)	0.003
Insulin	0.99 (0.53–1.84)	0.96	0.27 (0.10–0.74)	0.01
Sulfonylureas	0.39 (0.14–1.08)	0.07	0.23 (0.07–0.74)	0.01
α-Glucosidase inhibitor	1.14 (0.56–2.33)	0.71	0.41 (0.15–1.09)	0.07

*For the long-term all-cause death, there were 42 deaths of all the 282 eligible patients*.

## Discussion

The main goal of this retrospective study was to analyze the clinical characteristics and outcome of death in patients with pulmonary TB and type 2 diabetes comorbidity. We found that (1) the patients who died had lower albumin and hemoglobin level, and higher CCI score at admission as compared with the survival patients; (2) patients in the survival group had a higher usage rate of metformin than patients who died; (3) the higher albumin level was the independently protective factor, but older age and CCI score ≥3 were the independent risk factors for 1-year all-cause death in patients with pulmonary TB and type 2 diabetes comorbidity; (4) the higher albumin level, the use of metformin, insulin, or sulfonylureas were the independently protective factors, but age and CCI score ≥3 were the independent risk factors for the long-term all-cause death. The study finds that nutritional supplements and using metformin, insulin, or sulfonylureas to manage blood sugar may be helpful to reduce the mortality in patients with pulmonary TB and type 2 diabetes comorbidity.

In our included patients, the proportion of males was significantly higher than that of females (80.50 vs. 19.50%), which was consistent with the previous study. A meta-analysis including 88 surveys with over 3.1 million participants in 28 countries showed that overall male-to-female prevalence ratios were 2.21 for bacteriologically positive TB and 2.51 for smear-positive TB, with strong evidence that men were disadvantaged in seeking and/or accessing TB care in many settings (13). The higher prevalence of TB in males may be associated with the underlying genetic predisposition, sex hormone levels, social division of labor, and living habits in different genders (14, 15). Moreover, the prevalence of diabetes in men was also higher than in women in China (11.7 vs. 10.2%) (16).

In our study, the higher albumin level was the protective factor, and CCI score ≥3 was the risk factor for all-cause death in patients with pulmonary TB and type 2 diabetes comorbidity. To some extent, the albumin level and CCI score can reflect the nutritional status and the severity of underlying diseases in patients. Similarly, Okamura et al. reported that hypoalbuminemia on admission was the predictive risk factor for in-hospital mortality in TB patients (17). But there was currently insufficient research to know whether nutritional supplements reduced the mortality in TB patients, but it probably improved weight gain in some settings (18). Initially, the CCI score was used to predict the risk of death in hospitalized patients within 1 year, but later studies found that the CCI score could also predict the prognosis of chronic diseases such as chronic obstructive pulmonary disease (COPD), autoimmune diseases, and tumors. In this study, the CCI score was first used to predict the prognosis for patients with pulmonary TB and type 2 diabetes comorbidity, and we found that the higher the CCI score of the patient, the higher the risk of death.

Surprisingly, we found that, similar to the recommended hypoglycemic drug of insulin, the use of metformin or sulfonylureas was also the independently protective factor for the long-term all-cause death in patients with pulmonary TB and type 2 diabetes comorbidity. For metformin, it can not only reduce the blood sugar, but also has a variety of pharmacological effects such as inhibiting tumor growth, regulating immunity, and delaying aging (19). Moreover, metformin can reduce the growth of Mtb in macrophages (20), promote Mtb killing in lung epithelial cells and macrophages (21), ameliorate chronic inflammation and lung pathology, and improve the control of Mtb (22). Further, previous TB infection can increase the risk of COPD (23), acute myocardial infarction (24, 25), and tumors (26), and metformin can improve the long-term prognosis of these diseases (27–29). For sulfonylureas, it is the inhibitor of acetohydroxyacid synthase, which has been regarded as a potential drug target against Mtb as it catalyzes the first step in the pathway for the biosynthesis of branched-chain amino acids of bacteria, algae, plants, and fungi (30). Wang et al. and Liu et al. reported that monosubstituted sulfonylureas exhibited a significant anti-tubercular activity against extensively drug-resistant Mtb (31, 32). Furthermore, in addition to the proposed properties of the drug, probably metformin is a surrogate marker for the shorter evolution of diabetes and better glucose control (same as sulfonylureas) compared to insulin treatment (33). So metformin and sulfonylureas may protect the prognosis of pulmonary TB. Future large-scale and high-quality prospective studies are needed to verify their protective effect in patients with pulmonary TB and type 2 diabetes comorbidity.

Some strengths of this study need to be highlighted. First, we obtained the abundant clinical parameters of all patients for analysis. Second, all patients were followed up for at least 3 years, and the majority of the patients have been followed up for 5 years. Third, we analyzed the impact of different types of diabetes medications on the death of patients with pulmonary TB and type 2 diabetes comorbidity. Nevertheless, there are several limitations in this study. First, this is a retrospective study. The nature of the retrospective study has a limitation to reach final conclusions. Second, the patients included in this study are hospitalized patients and many patients have underlying diseases, so the conclusions of this study may not be applicable to the general population in the community. Third, TB drug resistance, anti-TB regimens, compliance with anti-TB treatment and cardiovascular drugs (such as statins), and other confounders of pulmonary TB were not taken into consideration.

## Conclusions

In conclusion, the lower albumin level, older age, and CCI score ≥3 were predictors of all-cause death in patients with pulmonary TB and type 2 diabetes comorbidity. In the long run, patients who use metformin, insulin, or sulfonylureas as hypoglycemic agents may have a lower incidence of death. Future large-scale and high-quality prospective studies are needed to verify the impact of nutritional supplements and different diabetes medications on the mortality of patients with pulmonary TB and type 2 diabetes comorbidity.

## Data Availability Statement

The raw data supporting the conclusions of this article will be made available by the authors, without undue reservation.

## Ethics Statement

The studies involving human participants were reviewed and approved by the Ethics Committee of West China Hospital of Sichuan University (2019-1103). Written informed consent for participation was not required for this study in accordance with the national legislation and the institutional requirements.

## Author Contributions

HF contributed to the conception and design of the research. SZ, XT, and LeW acquired the data from the database of West China Hospital of Sichuan University in China. SZ and XT performed statistical analyses. SZ, LeW, XT, JH, DW, LiW, and TZ drafted the manuscript. All authors contributed to the article and approved the submitted version.

## Conflict of Interest

The authors declare that the research was conducted in the absence of any commercial or financial relationships that could be construed as a potential conflict of interest.

## Publisher's Note

All claims expressed in this article are solely those of the authors and do not necessarily represent those of their affiliated organizations, or those of the publisher, the editors and the reviewers. Any product that may be evaluated in this article, or claim that may be made by its manufacturer, is not guaranteed or endorsed by the publisher.
